# Telehealth Interventions in Pharmacy Practice: Systematic Review of Reviews and Recommendations

**DOI:** 10.2196/57129

**Published:** 2025-05-07

**Authors:** Rachel Lai Kay Chong, Andrew Siang Ee Chan, Crystal Min Siu Chua, Yi Feng Lai

**Affiliations:** 1 NUS Pharmacy and Pharmaceutical Sciences Singapore Singapore; 2 MOH Office for Healthcare Transformation Singapore Singapore

**Keywords:** pharmacy, telehealth interventions, systematic review of reviews, recommendations, telehealth, telepharmacy, pharmacy practice, innovation, systematic review, Singapore, Intervention, service, literature, database, teleconsultation, telemonitoring, telecollaboration, telesupport, meta-analysis, telephone, fax, electronic messaging, electronic records, video conferencing

## Abstract

**Background:**

Pharmaceutical care has expanded, with telehealth playing a key role, especially during the COVID-19 pandemic. Despite global growth, existing reviews focus on specific settings or conditions, highlighting the need for broader research on public health topics and comparative studies to evaluate the effectiveness, preferences, and cost of telehealth interventions in pharmacy practice.

**Objective:**

The aim of this study was to unify existing literature on the impact of telehealth on future pharmacy practice and to analyze those already implemented in current pharmacy practice, with the objective of providing recommendations.

**Methods:**

The PRISMA (Preferred Reporting Items for Systematic Reviews and Meta-Analyses) framework was used to guide this review. In total, 4 databases were searched for relevant studies: PubMed, CINAHL, Web of Science, and Cochrane Library. Title, abstract, and full-text screening was performed, and 18 reviews met the selection criteria. The search period was from August 1, 2012, to December 22, 2024. The quality of the reviews was assessed using a 5-point Likert scale and a GRADE-CERQual scale.

**Results:**

Based on the identified reviews, telehealth interventions were categorized into teleconsultation, telemonitoring, telecollaboration, and telesupport. Teleconsultation was the most frequently used. Telephones were most common in teleconsultations and telemonitoring, while mobile, web, or computer applications were most frequent in telesupport. A combination of methods was most used to facilitate telecollaboration, such as telephone, fax, electronic messaging, shared electronic records, and videoconferencing. The identified reviews were evaluated by health outcomes, hospital readmission rates, patient safety, adherence, satisfaction, pharmacist shortage, and quality and access to care. The use of telehealth in pharmacy has generally seen an improvement in overall outcomes compared to traditional pharmacy practice. Our results show a strong push to integrate telehealth into future pharmacy practice, with the United States leading the way in adoption, demonstrating increased care access, quality, and patient safety. In Singapore, telephone consultations have been commonly used in hospitals, though community settings lack widespread adoption. However, the growing digital literacy of older adults and innovations like chatbots and telemonitoring present opportunities to expand telehealth services. To align with this shift, pharmacy education should invest in enhancing formative training by incorporating telehealth training, ensuring future pharmacists are prepared for this evolving practice, applicable to regions with similar contexts.

**Conclusions:**

Telehealth has shown promise in improving overall outcomes in pharmacy practice. While many countries have made strides, particularly in hospital settings, there remains an opportunity for greater adoption in community health care, driven by innovations like telemonitoring and digital literacy among older adults. The findings from this study can be used to inform future implementation of telehealth interventions in pharmacy in Singapore and other regions or cities with similar contexts.

## Introduction

Pharmaceutical care is an essential component of the health care system and has seen a significant expansion of roles and responsibilities in recent years to accommodate the evolving demands of patients [1]. Delivery of pharmaceutical care has been moving toward a more telehealth-focused approach, with pharmacists depending on communication technologies to communicate with both patients and other health care professionals, especially with the onset of the COVID-19 pandemic [2]. As such, many countries, including Singapore, have recognized the need to incorporate telehealth solutions to manage patient care, particularly considering pandemic-related pressures on health care systems. Telehealth is broadly defined as the use of communication technologies to deliver health care services remotely, encompassing a variety of services such as teleconsultations, remote monitoring, and virtual health support [2].

In 2019, Singapore’s Multi-Ministry Taskforce implemented telehealth to prevent overloading critical primary and secondary health care institutions during the pandemic. Considering these challenges, the Taskforce launched a series of initiatives aimed at reducing the strain on hospitals and clinics by expanding the use of telehealth. This proactive shift toward telemedicine allowed health care providers to continue offering essential services while maintaining social distancing measures and reducing the physical presence of patients in health care settings. As part of this initiative, the Telemedicine Allocation and Reconciliation System was also used to manage teleconsultation assignments to a suitable telemedicine provider, storing patient records and flagging patients who require additional care [3]. Additionally, the Ministry of Health has launched a regulatory sandbox initiative, providing impetus to startups to explore telemedicine with the support from the ministry. Platforms generated because of this initiative, such as MyDoc and DoctorAnywhere, have enabled greater access to teleconsultations, especially during the COVID-19 pandemic [4]. These telehealth platforms, alongside the regulatory sandbox, empowered not only private sector innovation but also allowed public health care institutions to explore new ways of delivering care.

Globally, similar trends have emerged, with many countries launching regulatory frameworks, innovation hubs, and collaborations with tech startups to foster telemedicine adoption. For example, in the United States, teleconsultations were used in 85% of major teaching hospitals, 49% of microrural hospitals, and 54% of nonprofit hospitals [5]. Similarly in India, an initiative by the Ministry of Health and Family Welfare launched digital outpatient services for all Indian citizens via the national Teleconsultation Service, eSanjeevaniOPD, expanding access to free health services to all its citizens [6]. The adoption of telehealth solutions has not only been a response to COVID-19 but also transformed how health care services are delivered [2]. As health care systems globally grapple with increasing patient demands, telehealth presents a sustainable solution to address gaps in care delivery, particularly in underserved and rural areas where access to health care professionals and pharmacists may be limited. As such, telehealth has become a cornerstone of modern pharmaceutical care, providing pharmacists with the tools to extend their services beyond traditional settings and reach patients wherever they are.

The shift toward telehealth in pharmacy practice has generated a growing body of literature and reviews [2]. However, many of these reviews tend to focus on specific settings or modes of telehealth, such as teleconsultations or telepharmacy in clinical environments. Additionally, many studies are limited to specific disease conditions and cover a limited range of public health topics, such as medication adherence. Hence, evaluation of a broader range of public health topics is required to provide a more holistic assessment of the benefits of telehealth interventions in the pharmacy setting. Moreover, further research in blinded comparable studies to investigate preference, appropriateness, and cost-effectiveness of telehealth interventions is required, which most current literature does not address.

To address these gaps, it is crucial to unify the currently available evidence regarding the four main telehealth domains: (1) teleconsultation, (2) telecollaboration, (3) telesupport, and (4) telemonitoring; their use in a variety of settings to identify future directions that pharmacy can possibly work toward [7]. Furthermore, there is a need for a more robust consolidation of existing information that explores the appropriateness of telehealth interventions in pharmacy practice that have been largely overlooked in existing research.

This systematic review of reviews was conducted to unify existing literature concerning the impact of telehealth on future pharmacy practice. The research question is as follows: What are the effective telehealth interventions in pharmacy practice, and what recommendations can be made? We hope to analyze the various telehealth interventions that have been implemented in current pharmacy practice and theorize recommendations on how these interventions may be applied.

## Methods

### Overview

The systematic review of reviews was conducted to synthesize existing evidence across multiple studies, offering a comprehensive understanding of a topic and highlighting trends and gaps in the literature. The methodology of this review was based on the PRISMA (Preferred Reporting Items for Systematic Reviews and Meta-Analyses) [8].

### Search Strategy

Standardized search terms will be used on 4 databases, PubMed, Cochrane Library, CINAHL, and Web of Science, from August 1, 2012, to December 22, 2024. The search terms were developed based on the keywords in our research question. These included telehealth and pharmacy practice, which were used to develop the search strategy: (tele* OR ehealth* OR digital*) and (pharmacy OR pharmacies OR pharmacist*) were used to search all the databases. MeSH (Medical Subject Headings) terms for “Telemedicine,” “Pharmacy,” “Pharmacies,” and “Pharmacists” were used in addition to the basic search terms in PubMed and Cochrane Library. “Telehealth+”, “Pharmacy Service+”, “Pharmacy, Retail,” and “Pharmacists” were additional MeSH terms used in CINAHL. The search was restricted to title, abstract (and keyword for the Cochrane Library). Only reviews published in English were included. The results were filtered for reviews, systematic reviews, meta-analyses, and meta-synthesis. The detailed search strategy is presented in Multimedia Appendix 1.

Closer to the end of the report and before publication, an updated search with the same search strategy was conducted to include any new reviews that have been recently published. No new articles of interest were found. PRISMA checklist can be found in Multimedia Appendix 2.

## Results

### Screening Process and Exclusion and Inclusion Criteria

**Figure 1:**
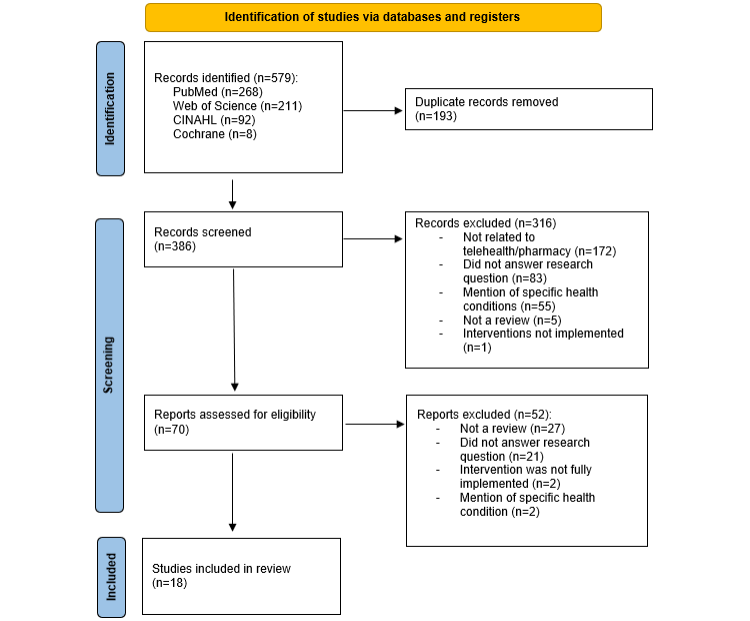
PRISMA (Preferred Reporting Items for Systematic Reviews and Meta-Analyses) diagram. Overall, 18 articles were included in this review.

Figure 1 shows the PRISMA diagram describing the review screening process. The database search returned 579 titles across the 4 databases, including duplicates. The results from each database were exported to EndNote to remove duplicates, and a manual search was conducted to ensure all duplicates were removed before proceeding to the screening process. Removal of duplicates resulted in a total of 386 reviews to be included in the screening, with 18 articles being included in this review. The PRISMA Checklist can also be found as Multimedia Appendix 2.

Screening of the title, abstract, and full text was conducted by authors 1 (RLKC) and 2 (ASEC), and any disagreement was resolved through discussion or through a third party, the last author (YFL). All identified reviews were screened for their relevance to the research question using the set of exclusion and inclusion criteria generated during each stage of the screening process (Table 1).

**Table 1:** Eligibility criteria for selecting articles on telehealth and pharmacy practice. Inclusion criteria: reviews written in English focusing on telehealth and pharmacy practice published within the last 12 years of relevancy. Exclusion criteria: non-review articles, published in languages other than English, focused on specific population or animal studies that were published more than 12 years ago.

Criteria	Inclusion criteria	Exclusion criteria
Article type	Reviews	Clinical trials, primary studies, editorials, letters to editor
Language	English	Other languages
Context	Telehealth and pharmacy practice	Unrelated to telehealth or pharmacy practice Focused on specific populations or animal studies
Time frame	Studies published within the last 12 years	Studies published more than 12 years ago

### Data Extraction and Analysis

Data from the included reviews were extracted for further processing and analysis in the final report. Data regarding study characteristics such as the year of publication, authors, objectives, interventions, discussions, and conclusions were extracted and summarized into Multimedia Appendix 3. Data analysis was conducted using the GRADE-CERQual.

### Principal Findings

Table 2 illustrates the proportion of telehealth interventions in the reviews. Notably, all combinations of interventions included teleconsultation, highlighting its prominence and extensive research. This table is also illustrated as a pie graph included as Multimedia Appendix 4

**Figure 2:**
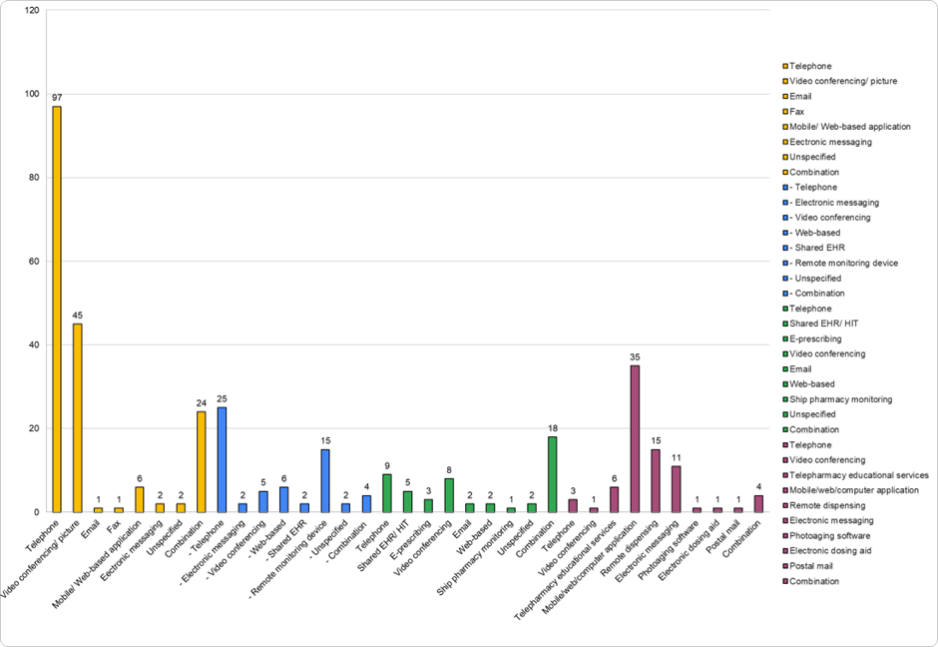
Telehealth intervention delivery methods in 18 reviews on telehealth in pharmacy practice. This bar graph illustrates the distribution of different delivery methods used in telehealth interventions across 18 review articles published between August 2012 and December 2024. Telephone-based care was the most widely used method, followed by mobile and web applications. The chart provides a breakdown of the various delivery methods used in teleconsultations, telemonitoring, telesupport, and telecollaboration. Common features associated with these interventions, such as improved health outcomes, reduced hospital readmissions, enhanced patient safety, better medication adherence, increased patient satisfaction, and greater access to care, are further summarized in Table 2. Additionally, the graph highlights the role of telehealth in addressing the pharmacist shortage by extending the reach of pharmacy services.

Telephone-based care was most common in teleconsultations and telemonitoring, while mobile, web, and computer applications were prevalent in telesupport. Telecollaboration typically used a mix of methods, such as telephone, fax, electronic messaging, shared EHR, and video conferencing (Figure 2).

Common features highlighted in the reviews are presented in Table 3. There was a general improvement in health outcomes, hospital readmission rates, patient safety, adherence, satisfaction with service provided, access to care, quality, and overcoming pharmacist shortage.

**Table 2:** Proportion of telehealth interventions in the 18 reviews on telehealth in pharmacy practice published between August 2012 and December 2024.

Intervention types		Reviews, %
Unrelated/pharmacist involvement unspecified		2
**Combination**		
	Telepharmacy (unspecified combination)	13
	Teleconsultation and monitoring	2.4
	Teleconsultation and support	2
	Teleconsultation and collaboration	0.2
	Teleconsultation, monitoring, and support	0.2
	Teleconsultation, monitoring, support, and collaboration	0.2
Telesupport		16.9
Telecollaboration		10.8
Teleconsultation		39
Telemonitoring		13.2

**Table 3:** Summary of intervention outcomes from telehealth services in pharmacy practice. This table summarizes the effects of telehealth interventions on health outcomes, including hospital readmissions, patient safety, medication adherence, satisfaction, access to care, quality of care, and pharmacist shortages. Telehealth generally improved surrogate markers and clinical outcomes, though results were mixed across studies. While patient satisfaction and medication adherence showed varied results, telehealth enhanced access to care and operational efficiency. Cost savings were noted in some areas, and telehealth helped alleviate pharmacist shortages, particularly in rural settings. +: improved; –: worsened; +/–: mixed results.

	Health outcomes			Hospital readmission		Patient safety				Adherence		Satisfaction		Time		Access		Pharmacist shortage		Quality		Cost
	Surrogate markers	Clinical outcomes			Med errors		ADR^a^	Drug safety														
Lowry et al [9]	+	No difference																				
Pathak et al [10]					+/–				No difference		–											
Sarkar et al [11]					+						+				+		+		+			
Baldoni et al [12]											+				+		+				+	
Crilly and Kayyali [13]	+	+									No difference				+							
Diedrich and Dockweiler [14]	No difference	No difference							+						+							
Emadi et al [15]									+/–													
Kane-Gill et al [16]	+	+													+				+		+/–	
Lobo Borba and Woranovicz Carvalho [17]															+							
Niznik et al [18]	+	+	+						+													
Park et al [19]	+/–	+/–	No difference		+		+		+													
Strnad et al [20]		+			+						+		+								+	
Unni et al [21]															+				–		–	
Andrzejewski et al [22]		+/–											–								+/–	
Baines et al [23]									+													
Dat et al [24]	+	+			–		+	+			+										+	
Lopez et al [25]		+	+		+																	
Melton et al [26]		+													+						+/–	

^a^ADR: adverse drug reaction.

### Health Outcomes and Hospital Readmission

Health outcomes remain the most widely discussed among the included reviews. Close to half of the reviews (n=8, 50%) either referenced improved surrogate markers or clinical outcomes or a combination of both in their discussions, while 2 revealed mixed results.

Surrogate markers like hemoglobin A_1c_, blood pressure, International Normalized Ratio (INR), and total cholesterol improved due to telehealth services. Crilly and Kayyali [13] highlighted that community pharmacists' telemonitoring services significantly improved home blood pressure control in the intervention group, which transmitted readings remotely at 6, 12, and 18 months, compared to the control group.

Clinically significant outcomes, such as disease control targets from telehealth interventions, were widely discussed. Strnad et al [20] reported improvements in antimicrobial stewardship through email, with daily recommendations from ID physicians and pharmacists reducing hospital-acquired *Clostridium difficile* infections and avoidable antimicrobial use. Additionally, there were improvements in bleeding risk for patients on anticoagulation. One review on telemedicine by clinical pharmacists in Colorado found reductions in major bleeding episodes and thromboembolic fatalities compared to standard care [16].

In total, 3 of the reviews reported an impact on hospital readmission rates with the use of telehealth interventions by pharmacists. According to two other reviews, telehealth enabled pharmacists to extend their reach to rural patients or those unable to be physically present, promoting better transitions of care post discharge [18,25]. Park et al [19] mentioned no difference in hospital readmission rate; however, this only pertained to patients with cancer.

### Patient Safety, Satisfaction, and Medication Adherence

In total, 6 reviews addressed telehealth services and their impact on medication errors, adverse drug reactions, and drug safety, all related to patient safety. In total, 4 of these reviews reported a reduction in medication errors with telehealth integration. Sarkar et al [11] found significant decreases in medication errors in emergency departments and patient medication regimes when barcode technology was used. Another review noted that telephone medication history interviews helped identify discrepancies in patients’ medical records, including missing information and medication errors. However, most reviews did not discuss the implications of telehealth on drug safety. Dat et al [24] highlighted that telepharmacy services promote drug safety and efficacy by preventing adverse side effects.

There was generally an increase in patient and nurse satisfaction with the use of telehealth in pharmacy practice. In total, 2 exceptions reported no difference in satisfaction, which stated that patient satisfaction with telepharmacies was lower or no different compared to traditional pharmacies, partly due to the longer time required to obtain counselling and medications through telepharmacies [10,13].

In total, 6 of the 18 reviews included in the final review examined medication adherence. The majority of these 6 reviews (n=4, 66.6%) observed improved medication adherence for patients who used telehealth services for their care. One review mentioned that the average usage technique of metered dose inhalers in patients with asthma increased significantly because of multiple 15-minute video consultations [14]. Similarly, telehealth services mentioned in Emadi et al [15], such as text, video, and telephone, greatly improved medication adherence in certain populations, specifically the older generation who receive care from remote rural clinics. However, Emadi et al [15] did note that these improvements were not consistent across all patient populations. Participants that maintained a high level of adherence before the initiation of telehealth interventions remained at the same level following its use. Moreover, patients with HIV and chronic pain also did not experience significant changes in medication adherence following the initiation of these interventions [15].

### Quality, Access to Care, and Operational Efficiency

One review indicated that telehealth improved the quality of care through increased medication action, therapy review, and monitoring performed by pharmacists [11]. Similarly, Kane-Gill et al [16] suggested that variability in treatments could be decreased due to increased frequency of follow-ups. However, Unni et al [21] reported a decrease in quality compared to traditional pharmacy practice, as certain assessment tools could not be modified to suit the telepharmacy setting. Additionally, Unni et al [21] reported that diminished patient engagement and increased dispensing errors were observed as reasons for the perceived reduction in the quality of care.

In total, 8 reviews highlighted that telehealth services either provided greater access to 24-hour pharmaceutical services, particularly in hospitals that do not have the capacity for such services or eliminated barriers such as travel distance. Health care professionals were able to consult pharmacists even during night shifts, which led to a greater number of therapeutic interventions [20]. A rise was also seen in the number of patient consultations and health screenings conducted through telehealth modalities [11,17].

There were mixed results regarding the effect of telehealth on operational efficiency. Strnad et al [20] reported a reduction in mean order processing time [20]. Conversely, Andrzejewski et al [22] found that asynchronous workflow and indirect communication led to longer intervention times, indicating that telehealth could sometimes delay service provision.

### Cost and Pharmacist Shortage

The impact of telehealth on the costs varied among reviews. Andrzejewski et al [22] and Kane-Gill et al [16] reported that cost savings resulted from preventing unnecessary antimicrobial use or medication errors and adverse drug reactions, while Melton et al [26] stated that costs related to lost wages and productivity due to traveling were prevented. Baldoni et al [12] also mentioned that a single remote pharmacist could cover several sites in place of multiple physical pharmacies, which contributed to lower costs of both manpower and infrastructure. However, greater costs may be incurred to acquire the infrastructure and technology required to perform telehealth or due to a lack of reimbursement policies in place [16,21,26].

Baldoni et al [12] and Sarkar et al [11] mentioned that telehealth helped to relieve the issue of pharmacist shortage, especially in rural hospitals, by allowing workload to be divided between onsite and offsite pharmacists, enhancing productivity and overall care for patients.

### Quality Assessment

A 5-point Likert scale risk assessment table was created to evaluate the quality of the included reviews, with 1 indicating “very unsatisfactory” and 5 indicating “very satisfactory.” The domains evaluated were (1) search strategy, (2) screening and selection of articles, (3) data extraction, and (4) data interpretation (Multimedia Appendix 5). Points were deducted based on reasons that the authors felt would reduce the quality of the review, such as the absence of a clear search strategy or screening process or the omission of information such as the type of data extracted. A more detailed breakdown can be found in Multimedia Appendix 6. Corresponding study number can be found in Multimedia Appendix 7.

For a review to have attained a maximum point score of 20, they would have had to meet the “very satisfactory” criteria for all 4 domains of its methodology. For reviews that possessed methodological limitations that were of no concern, they attained scores between 17 and 20. Those with methodological limitations that were of minor or moderate concern had scores between 13 and 16 and 10 and 12, respectively. From Table 4, of the 18 included reviews, 15 reviews had methodological limitations of no concern, 2 had minor concerns, and 1 had moderate concern.

**Table 4:** Quality assessment table. This table presents the evaluation scores for each article across 4 key categories: search strategy, screening and selection of articles, data extraction, and data interpretation. Most reviews that possessed methodological limitations that were of no concern attained scores between 17 and 20.

Article	Search strategy	Screening and selection of article	Data extraction	Data interpretation	Total
Lowry et al [9]	5	5	5	3	18
Pathak et al [10]	5	4	5	4	18
Sarkar et al [11]	5	4	4	5	18
Baldoni et al [12]	5	5	5	5	20
Crilly and Kayyali [13]	5	4	5	4	18
Diedrich and Dockweiler [14]	4	4	5	1	14
Emadi et al [15]	5	5	5	3	18
Kane-Gill et al [16]	5	5	4	3	17
Lobo Borba and Woranovicz Carvalho [17]	5	4	5	4	18
Niznik et al [18]	5	5	5	5	20
Park et al [19]	5	5	5	5	20
Strnad et al [20]	3	5	5	5	18
Unni et al [21]	3	5	4	5	17
Andrzejewski et al [22]	1	1	5	5	12
Baines et al [23]	5	5	5	5	20
Dat et al [24]	5	5	5	5	20
Lopez et al [25]	4	4	5	3	16
Melton et al [26]	5	5	5	5	20

Additionally, a GRADE-CERQual scale was used to evaluate the amount of confidence to place in the review findings of the included reviews. Common findings in reviews were grouped together and evaluated according to adequacy, coherence, and relevance and were categorized as either no concerns, minor concerns, or moderate concerns. The majority of the findings had either no or minor concerns. A more detailed breakdown can be found in Multimedia Appendices 6 and 7.

## Discussion

### Overview

Overall, the results segment has shown that telehealth enhanced care quality and access but had mixed effects on operational efficiency, with potential for cost savings and alleviating pharmacist shortages despite initial infrastructure costs. Telehealth has been increasingly acknowledged as a stable alternative to usual care provided by onsite pharmacists, especially since the advent of the COVID-19 pandemic. Many factors account for this trend, including the ability of telehealth to provide pharmaceutical care across big distances, thereby increasing access to health care [27]. Additionally, manpower shortages can be bridged by connecting remote rural hospitals to larger main pharmacies [28]. Besides this, there has been a shift in focus to a more multidisciplinary approach to patient care in recent years, increasing cooperation between health care professionals, which is facilitated by telehealth [29]. Hence, pharmacy practice has been seeing a rise in the use of telehealth modalities, which is likely to continue in the future.

However, there are certain challenges that limit the growth of telehealth utilization in pharmacy practice. This includes regulations, infrastructure, and technological costs or lack of evidence to support use. Moreover, many studies have highlighted the need to provide pharmacists with adequate training and education to operate telehealth-associated technology and adapt counseling methods or tools to suit telepharmacy before such interventions may be properly implemented into practice [15,16,21,24].

### Changing Roles of Pharmacists

Globally, pharmacists have seen a shift away from the traditional roles of dispensing medications toward more patient counseling and monitoring [1]. There has been increasing recognition of pharmacists as an underused group of health care providers who have the necessary skills and knowledge to manage patients in the community setting. This was partly due to the COVID-19 pandemic, whereby many hospitals were overloaded with the surge in cases, resulting in a greater dependence on pharmacists to handle less urgent medical needs. Additionally, there has been a rising prevalence of chronic conditions, such as diabetes or hypertension, that require routine follow-ups and may be adequately managed in the community. Hence, pharmacists are in an ideal position to help patients with chronic conditions or minor ailments while relieving the burden on other health care professions.

Meanwhile, with the rising focus on the significance of multidisciplinary health care and the ease of communication through technology, more telecollaboration was expected. However, telecollaboration consisted of only 11.2% of the interventions in our identified reviews, including telecollaboration alone and in combination with other telehealth interventions. This may be due to traditional physical interactions between health care providers still taking precedence over communicating via virtual means whenever feasible, especially if the health care providers are within proximity of each other.

### Significance of Certain Modes of Telehealth Delivery

Telephones are one of the most accessible and inexpensive forms of communication, which supports their extensive use in teleconsultations and telemonitoring. This typically involves verbal discussions between pharmacists and their patients; thus, telephones tend to be a sufficient means of communication.

Internationally, there has been a significant focus on protecting the privacy and security of individuals' health information [20]. For instance, in the United States, there has also been a rise in Health Insurance Portability and Accountability Act–compliant video conferencing platforms, including Zoom and Skype [21]. This was mostly a result of the COVID-19 pandemic when inexpensive platforms were needed urgently to facilitate the delivery of health care services despite social distancing measures in place. Therefore, increased accessibility to such video conferencing platforms likely enabled greater uptake by pharmacists to communicate with both patients and other health care professionals as seen in our results.

The reviews revealed an unexpected finding: the frequent use of mobile, web, and computer applications in telesupport. Despite older patients often struggling with new technology, both patients and health care providers showed receptiveness to these applications. The COVID-19 pandemic accelerated their adoption in health care institutions to reduce disease transmission while maintaining access to services. Thus, this could have influenced patients to be more receptive to such health applications to reduce the risk of COVID-19 infection. Besides this, health applications provide patients with greater access to their health records, which potentially empowers them to gain a better understanding of their condition and engage them in more informed discussions with their health care providers [30]. This feeling of empowerment could have contributed to greater uptake of health applications by patients as well [31,32].

Conversely, remote monitoring devices were expected to be the most used in telemonitoring due to their close association, which was not the case in our findings. A possible explanation would be the additional costs posed by remote monitoring devices. Electronic dosage forms that track adherence, such as blisters, eye drops, or sprays, may be more expensive than regular dosage forms, thus deterring uptake [15]. Moreover, remote monitoring devices may be unavailable in many areas, leading to telemonitoring being conducted through more accessible means like telephones.

### Implications and Recommendations

From our results, we observe a growing international effort to incorporate telehealth into future pharmacy practice. For example, the United States became the forerunner for adopting telehealth modalities into its practice. This shift has resulted in increased access to care, improved quality of care, enhanced medication adherence, and greater patient safety, all of which have proven the effectiveness of telehealth in pharmacy practice. These successes have provided strong motivation for other countries to follow suit and explore the potential of telehealth to improve health care delivery.

On a global scale, many countries have leveraged telehealth solutions to overcome challenges such as access to care in remote areas, health care workforce shortages, and the pressures of aging populations [11]. In regions like Europe, Australia, and Canada, telehealth adoption has rapidly expanded in both urban and rural settings, improving continuity of care, especially during public health crises such as the COVID-19 pandemic [21,24].

Locally, in Singapore, telephone consultations have been regularly used by hospitals. For example, in local hospitals, telephone consultations acted as alternatives for follow-ups, especially for outpatient care clinics. The COVID-19 pandemic, coupled with Singapore’s aging population, drove the inception. However, such methods are rarely seen in the community setting. As community general practitioner clinics act as points of confluence for neighborhood residents, incentive for teleconsultation has lacked considerably. In 2012, Guardian introduced MyDoc, an application for patients to interact with a panel of health care professionals, which includes both pharmacists and physicians [4]. With private community pharmacies taking the first steps toward telehealth integration into practice, efforts to replicate their success to allow for greater patient usage are no longer a hopeful belief [31]. Therefore, there lies a great opportunity for telehealth adoption in community health care in Singapore and regions and cities with similar geographical contexts.

With the older adult population having more digital literacy, there is increasing receptiveness to telehealth modalities such as telesupport and telemonitoring [15]. Chatbots can provide telehealth services is one of the opportunities to be explored. Chatbots can be used to provide educational services to aid patients in understanding their disease conditions [33]. Additionally, recording blood pressure and glucose levels among other monitoring parameters can enable greater monitoring by physicians and pharmacists to improve patient disease control [34]. Thus, telesupport and telemonitoring can serve as gateways for adopting changes in traditional pharmacy practice.

As telehealth services are increasingly introduced, pharmacy education must keep pace with the changing landscape and incorporate telehealth as a component of teaching and assessment, such as by conducting virtual formative assessments. This is supported by the identified reviews, which suggest that adequate training is required for pharmacists to deliver telehealth services due to its differences compared to traditional face-to-face interaction with patients [15,16,21,24]. Future pharmacists should be prepared to perform telehealth services competently, and a way to assure that would be through implementing skills and knowledge required for telehealth into undergraduate education. While some courses may touch on telehealth, this approach guarantees that future pharmacists are fully equipped for this evolving field. This can be applied to cities or regions with similar geographical context.

### Limitations

A limitation of this review is the subjectiveness of the quality assessment conducted on the 18 identified reviews. Although GRADE-CERQual is a widely used systematic and transparent approach to assess the quality of results in the identified reviews, it is still highly dependent on the authors’ opinions and judgment.

Moreover, a few identified reviews did not elaborate on the specific modes of delivery of telehealth interventions, making it challenging to classify them. This included one review that labeled the delivery modes as “telepharmacy services,” which we classified as a combination of delivery methods based on the context of the review. Additionally, 5 of the reviews had unspecified delivery modes that we were unable to classify into specific telehealth interventions. Therefore, since certain delivery modes were not explicitly stated, the current classification may not be entirely accurate. Furthermore, this review of reviews did not include gray literature, which may have resulted in the exclusion of potentially significant studies. Future reviews could benefit from incorporating gray literature to enhance the comprehensiveness of the findings.

Lastly, the research questions and search strategies in the identified reviews lack homogeneity. Although we screened these reviews, some may not fully relate to our research question, resulting in only a portion of the extracted information being relevant for our systematic review. This led to the exclusion of much irrelevant data. For example, Baldoni et al [12] met our inclusion criteria, but some data on antineoplastic medication preparation and the control of medicine chests on seagoing vessels were irrelevant to our research question.

### Conclusions

In conclusion, telehealth interventions have been increasingly established worldwide, demonstrating the potential to deliver both health and non–health-related outcomes in pharmacy practice. While many countries have made strides, particularly in hospital settings, there remains an opportunity for greater adoption in community health care, driven by innovations like telemonitoring and digital literacy among older adults. Community pharmacists can also be positioned as first responders to many health concerns. Additionally, integrating telepharmacy content into pharmacy curricula will better prepare future pharmacists for this evolving practice. The findings from this study can inform the future implementation of telehealth interventions in pharmacy.

## Data Availability

The datasets generated or analyzed during this study are available from the corresponding author on reasonable request.

## Appendix

Multimedia Appendix 1 Detailed Search Strategy.

Multimedia Appendix 2 PRISMA Checklist.

Multimedia Appendix 3 Data Extraction.

Multimedia Appendix 4 Proportion of telehealth interventions in the 18 reviews on telehealth in pharmacy practice. This pie chart illustrates the distribution of different types of telehealth used in 18 reviews, published between August 2012 and December 2024. Telephone-based care was the most common intervention, primarily used in teleconsultations and telemonitoring. Mobile, web, and computer applications were most prevalent in telesupport services. Telecollaboration, which typically involved a combination of telephone, fax, electronic messaging, shared electronic health records, and video conferencing, was used in several studies to enhance communication between health care providers. These interventions were applied in a variety of pharmacy practice settings, including community pharmacies and telepharmacy services.

Multimedia Appendix 5 GRADE-CERQual Table.

Multimedia Appendix 6 Reasons for Deduction of Scores (Risk Assessment).

Multimedia Appendix 7 Corresponding Study Numbers for GRADE-CERQual Assessment.

## Abbreviations

PRISMA: Preferred Reporting Items for Systematic Reviews and Meta-Analyses
MeSH: Medical Subject Headings
