# Supplementary material for: Telehealth Interventions in Pharmacy Practice: Systematic Review of Reviews and Recommendations
Source: J Med Internet Res. 2025 May 7;27:e57129. doi: 10.2196/57129 (PMC12096025; doi:10.2196/57129)
Supplement: Multimedia Appendix 1 [file jmir_v27i1e57129_app1.docx]

**Multimedia Appendix**

Multimedia Appendix 1. Detailed search strategy

| **No.** | **Database** | **Search Terms** | **Results**  **Total (excluding duplicates) = 386** |
| --- | --- | --- | --- |
| 1 | Pubmed | #1: "Telemedicine"[Mesh] OR tele*[tiab] OR ehealth*[tiab] OR digital*[tiab]  #2: "Pharmacy"[Mesh] OR "Pharmacies"[Mesh] OR "Pharmacists"[Mesh] OR pharmacy[tiab] OR pharmacies[tiab] OR pharmacist*[tiab]  #1 AND #2 | 268  (review, systematic review, meta analysis) |
| 2 | Web of Science | #1: (tele* OR ehealth* OR digital*) (Title) or (tele* OR ehealth* OR digital*) (Abstract)  #2: (pharmacy OR pharmacies OR pharmacist*) (Title) or (pharmacy OR pharmacies OR pharmacist*) (Abstract)  #3: #1 AND #2 | 211  (review) |
| 3 | Cochrane | #1 ( pharmacies OR pharmacy OR pharmacist*):ti,ab,kw  #2 MeSH descriptor: [Pharmacy] explode all trees  #3 MeSH descriptor: [Telemedicine] explode all trees  #4 (tele* OR ehealth* OR digital*):ti,ab,kw  #5 MeSH descriptor: [Pharmacies] explode all trees  #6 MeSH descriptor: [Pharmacists] explode all trees  (#1 OR #2 OR #5 OR #6) AND (#3 OR #4) | 8  (review) |
| 4 | CINAHL | S1: TI ( tele* OR ehealth* OR digital*) OR AB ( tele* OR ehealth* OR digital*)  S2: TI (pharmacy OR pharmacies OR pharmacist*) OR AB (pharmacy OR pharmacies OR pharmacist*)  S3: (MH "Telehealth+")  S4: (MH "Pharmacy, Retail")  S5: (MH "Pharmacists")  S6: (MH "Pharmacy Service+")  S7: (S1 OR S3) AND (S2 OR S4 OR S5 OR S6)  **meta analysis**  S8: (S1 OR S3) AND (S2 OR S4 OR S5 OR S6)  **meta synthesis**  S9: (S1 OR S3) AND (S2 OR S4 OR S5 OR S6)  **review**  S10: (S1 OR S3) AND (S2 OR S4 OR S5 OR S6)  **systematic review**  S11: S7 OR S8 OR S9 OR S10 | 92  (review, systematic review, meta analysis, meta synthesis) |
