# Supplementary material for: Telehealth Interventions in Pharmacy Practice: Systematic Review of Reviews and Recommendations
Source: J Med Internet Res. 2025 May 7;27:e57129. doi: 10.2196/57129 (PMC12096025; doi:10.2196/57129)
Supplement: Multimedia Appendix 3 [file jmir_v27i1e57129_app3.docx]

Multimedia Appendix 2. Data extraction

| **Authors/Year** | **Objectives** | **Interventions** | **Discussions** | **Conclusions** |
| --- | --- | --- | --- | --- |
| S. J. Lowry, C. N. Kay, E. N. Marsom, J. S. Park, S. Poole, A. T. Page (2020) | To assess if pharmacist-led telehealth interventions alter surrogate markers of health conditions. | Telephone consultation.  At least one adjuvant such as home monitoring, web-based portals, text message and written materials.  Other pharmacist telehealth interventions also comprised recommendations for medicine optimisation to the primary care practitioner | - Telehealth interventions had similar health outcomes for surrogate markerscompared to usual care   **-ve:**   - Extent of pharmacist involvement in participants’ usual care unclear - Did not explore videoconferencing - Studies were conducted to test the superiority of pharmacist services provided via telehealth rather than testing whether it was an appropriate alternative using a non-inferiority methodology - Telehealth is that it is dependent on the skills and attitude of the consumer when using technology | Telehealth interventions provides an acceptable adjunct to usual care and results in similar health outcomes for  surrogate markers when compared to usual care. |
| S. Pathak, C. M. Blanchard, E. Moreton, B. Y. Urick (2021) | How structural differences in the delivery of telepharmacy services affected process and outcome measures of quality in the community pharmacy setting.  Address gaps in current peer reviewed literature. | Telepharmacy services that include remote dispensing services and may or may not include remote patient counselling by a community pharmacist | - Some indication that telepharmacies performed comparably well or slightly better in catching medication-related errors and maintaining patient adherence to medications. - Telepharmacies seemed to perform slightly less well than or no differently on patient satisfaction. - Telepharmacy services can improve access to pharmaceutical care for people living in high-need communities | Scarce literature and high risk of bias, thus no definitive conclusions could be made about patient safety and quality-related outcomes for telepharmacies in the community pharmacy setting |
| R. Sarkar, B. J. Metzger, H. M. Sayre, C. M. Slater, S. Katamneni and A. Coustasse (2018) | To examine the effect of the use of telepharmacy on rural hospitals’ access to pharmaceutical services. | Telepharmaceutical activities in a single hospital or a small number of hospitals in an area | - Access: rise in on-call consultation and patient counseling sessions - Time: decreased time for medication order entry, time for locating medications and processing orders and turnaround time on patient order entries and checks on medications. - Errors: improved in the medication supply system and enhanced documentation of medications were achieved, reduced underdosing of drugs and erroneous patient medication histories - Pharmacist shortage: electronically transmit medication orders to a central pharmacy, improved the quality of care by monitoring other activities, distributing the workload between on-site and remote pharmacists - Quality: errors in the consumer’s pharmaceutical history were curtailed, barcode technology that improved accuracy in preparing chemotherapeutic drugs.   **-ve:**   - hamper the business of traditional pharmacies - fear of commercialisation of telepharmacists that will undermine pharmacists role | Telepharmacy may enhance access to pharmaceutical services for hospitals in rural areas that do not offer 24-hour pharmaceutical services |
| S. Baldoni, F. Amenta and G. Ricci (2019) | The present work has analysed the main studies reporting telepharmacy experiences with particular attention to those in some way different to the conventional pharmaceutical service. | Telepharmacy services already developed include medication selection, order review and dispensing, patient counselling and monitoring, and provision of clinical service.. | - Access: advances in technology may allow to reduce inequalities in healthcare delivery - Time: patients to save money and travel time, especially by old people, to reach healthcare structures. - Costs: reduction of pharmacy services costs as one pharmacist can cover multiple sites   **-ve:**   - Depends on extent of level of technological infrastructures, such as efficient internet connections - Protection and encryption of this health data to avoid data leaking. - Acquisition of software and devices may be a financial burden for small rural healthcare. - Degree of scepticism of both health practitioners and patients. | Positive evaluation of telepharmacy services and encourage their development primarily to counter pharmacist shortage. |
| P. Crilly and R. Kayyali (2020) | To understand how pharmacists have used telehealth and digital technologies to improve public health related outcomes, addressing NON COMUNICABLE DISEASES as well as communicable diseases and medication-related topics. | The majority of the studies used telephones as their intervention tool. while only one study used an mHealth app, two studies used a tele-monitoring device and one study used photo-aging software. | - Medication Adherance: telephone-based pharmacy advisory service had a significant impact in reducing non-adherence to medication, an mHealth app to improve adherence in adolescent asthmatics, but did not affect patient quality of life or asthma control. - Patient knowledge: antidepressant study showed an increase in patient knowledge and medication beliefs - Drug related problems: telephone intervention was effective in identifying and managing drug related problems - Satisfaction: no differences in patient satisfaction with the information provided versus usual care. - Surrogate markers: significant improvements in BP control   **-ve:**   - no studies used social media as an intervention tool - The lack of use of video conferencing technology - limited use of “novel” technology by community pharmacists. - Digital literacy of the pharmacy workforce could be a key issue holding back the use of these tools. | There were limited studies on the use of more novel technology, with only one study using a mobile health app, one study using a remote health monitoring device, and no studies using social media. In addition, medication adherence was the main public health topic addressed in the studies, with other public health issues such as overweight and obesity, mental health, and sexual health not being investigated at all. |
| L. Diedrich and C. Dockweiler (2021) | The aim of this article is to investigate the effects of synchronous video-based teleconferences between pharmacists and patients or other service providers on patient-related outcomes compared to standard care. | In Choudhry et al. all received individualized telephone consultations with a pharmacist, additional forms of support were offered, with video-based teleconsultations being the most intense assistance.  Ishani et al. study included remote monitoring of relevant parameters and remote consultations with various service providers.  León et al. integrated telemedical aspects into an online patient monitoring system, which also included remote consultations as well as chat messages from health care providers and online education opportunities.  The study by Bynum and colleagues, investigates telemedical guidance of patients on the correct use of metered dose inhalers for the treatment of asthma. | - Scarce improvement in clinical and psychological outcomes - Adherance: the intervention in Choudhry et al. and Bynum et al.led to a improvement in adherence. León et al. found no significant difference in adherence. - Readmission: no difference in hospital readmission. Choudhry et al. found a significant decrease in emergency department visits in the intervention group. | Since the available results do not provide indications for an improvement of patient-relevant outcomes, a comprehensive implementation does not seem feasible in countries with already sufficient pharmaceutical care. Further studies are necessary to ensure fast and practical implementationCost-effectiveness should be a primary focus in order to achieve a successful and beneficial implementation. |
| F. Emadi, A. Ghanbarzadegan, S. Ghahramani, P. Bastani and M. T. Baysari (2022) | The current scoping review investigated the key factors that affect the medication adherence (MA) in older adults using tele-pharmacy interventions. | All tele-pharmacy services. Various methods of remote communication were identified, including electronic medical device, mobile application, web application, text message, postal mail, phone call,electronic-prescription (e-prescription) and remote dispensing. | - Older patients, particularly those with chronic diseases, demonstrated good MA when using tele-pharmacy services. - Facilitate communication between patients and health professionals would make interventions more effective - MA was higher in older groups (over 65 years) than in younger groups (less than 65 years) when using tele-pharmacy interventions - Many smartphone apps, particularly those that include interactive features, have been effective in improving MA for older patients. | Tele-pharmacy can improve MA in older people, however, several factors impact the achievement of this goal. Communication with the health care team and sufficient user training appear necessary for tele-pharmacy interventions to enhance MA. Government and policymakers should provide subsidies for such interventions to encourage uptake among older users. |
| S. L. Kane-Gill, J. D. Niznik, J. A. Kellum, C. M. Culley, R. D. Boyce, Z. A. Marcum, H. He, S. Perera and S. M. Handler (2017) | To determine what telemedicine services are provided by pharmacists in the nursing facility and their impact on resident care. |  | - Improve prescribing and offer potential patient safety and financial benefits, but are limited to settings largely outside of the nursing facility. - Cost savings from preventing medication errors and adverse drug events (ADEs). - Eliminate distance barriers, improve access to medical services - Treatment variability can be reduced. - Building better patient rapport over telephonic services by putting a face with the voice.   **-ve:**   - up-front costs associated with buying the telemedicine equipment and training the users. | There is sufficient evidence on the effectiveness of telemedicine, and future research is needed on implementation and practice-based methods. The current state of telemedicine is focused on increasing access to medical services. However, the benefits of telemedicine are shifting and now include an emphasis on reducing health care costs. |
| H. H. Lobo Borba and D. M. Woranovicz Carvalho (2022) | To investigate the current evidence concerning the impact of the 4IR (4th industrial revolution) on the provision of pharmaceutical services. | All the 59 included studies addressed technologies within the scope of telepharmacy, and a few articles detailed the type of technology applied, comprising Bluetooth, e-mail or chat, mobile applications or web-based applications, and videoconferencing softwares. | - Access: improved access by using automated drug dispensing system (ADDS) units and webcam technology, increased access to screening and improve diseases management Webcam-enabled videoconferencing allows effective interactions between pharmacists and patients to identify medication-related problems (MRPs) - Safety: enrich drug dispensing safety in pharmacies   **-ve:**   - lack of access for the population in many areas of developing countries. | The pandemic accelerated the use of technologies that have been shown to bring more agility to care and more impact on the population, identifying promising niches for pharmacists in the field of Pharmaceutical Care. |
| J. D. Niznik, H. He and S. L. Kane-Gill (2018) | To assess the impact of clinical pharmacist interventions on clinical outcomes, specifically clinical disease management, patient self-management, and adherence outcomes in outpatient or ambulatory care settings, when delivered using telemedicine. | Telemedicine technologies utilized for the delivery of interventions, including telephone, video consultation, text or electronic messaging, e-mail, automated electronic reports, and fax. | - Most studies reported an overall positive impact on outcomes. - Practical applications of outpatient telemedicine include reaching out to caregivers unable to physically meet with clinicians, to ensure better transitions of care or extended patient outreach uncommon in rural areas.   **-ve:**   - Studies focused on productivity, cost-effectiveness, and qualitative measures were not assessed. - While telemedicine technologies like internet/cloud services with video conferencing have been used for remote interventions to rural hospital patients, future study is needed of the use of such technologies, compared to phones only in outpatient settings. | Clinical pharmacy services delivered via telemedicine in the outpatient or ambulatory care setting, primarily via phone, have an overall positive impact on outcomes related to clinical disease management, patient self-management, and adherence in the management of a variety of chronic diseases. |
| T. Park, J. Muzumdar and H. Kim (2022) | The purpose of this study was to systematically review Clinical pharamcists-led digital interventions to improve patients’ health-related clinical outcomes. | Telephone use, web-based tool and a mobile app. | - Digital interventions generally lowered health service use and improved drug-related outcomes. - Did not always result in significant improvements in lab values, adherence, survival, and health-related risk. - Both clinical and community pharmacists used telephones most commonly for interventions. More novel technologies can be considered. - Community pharmacists’ interventions focused primarily on improvement in medication counseling and adherence, which represents traditional roles of a community pharmacist. Using more diverse technologies could help define their roles for more diverse outcomes/domains. - Little investigation into the cost-effectiveness. | Impacts of telephone-based interventions on patients’ outcomes were not consistent. Impacts of web-based interventions and mobile apps were generally positive, which suggests the benefits of continued use of these tools. future research is needed to first identify the economic value of the interventions and then implement the cost-effective interventions. |
| K. Strnad, B. R. Shoulders, P. L. Smithburger and S. L. Kane-Gill (2018) | To broadly define the effect, highlight opportunities for pharmacists, and compare the different services offered in both general wards and ICUs. | Telepharmacy staff received information either by remote access to the electronic medical record, faxed or scanned documents, picture/webcam, or some combination. Communication between telepharmacists and institutional personnel was conducted either by email/electronic communication or facsimile, video/picture review, telephone, some combination. | - Telepharmacists in the ICU setting provided overnight coverage. In the non-ICU setting, telepharmacists improved patient outreach, particularly to remote areas. - Off-site pharmacists are able to free up on-site pharmacists to complete other tasks. - Both indirect and direct cost savings were gained through telepharmacy use.   **-ve:**   - Purchasing and implementing the necessary technology. But not apparent because the studies reviewed predominantly relied on remote access to the electronic health records, and communication was primarily by fax, email, or telephone. - Concern of malpractice - Violation of pharmacy law. Currently, few institutions use telepharmacy despite clinical, operational, safety, emotional, and financial benefits. In most cases, only relatively basic technology has been implemented. | Technology and communication methods were similar in both ICU and non-ICU settings and assisted with the expansion of services in the former and improved patient outreach, particularly to remote areas in the latter |
| E. J. Unni, K. Patel, I. R. Beazer and M. Hung (2021) | To summarize the implementation of telepharmacy during the surge of COVID-19. | Communication technology (online or text), mobile application, videoconferencing and audio consultations, hotline, mail, fax, email. | - Lack of access to digital devices by individuals who would benefit the most from telepharmacy. - Need for educating and credentialing pharmacists in telepharmacy and in disaster management. - Further studies are needed to examine the reimbursement for telepharmacy consultations, impact of telepharmacy on patient safety and outcomes, workload, morale and attrition on pharmacy staff, public perception and access of technology, implementation of telepharmacy via social media. | COVID-19 pandemic increased the use of telepharmacy, but posed challenges. However, steps can be taken by pharmacy organizations, payers, and pharmacy entrepreneurs in leveraging the convenience of telepharmacy. |
| C. Andrzejewski, E. K. McCreary, T. Khadem, R. C. Abdel-Massih and J. R. Bariola (2021) | To explain the needs and challenges of building and sustaining inpatient ASPs, review the available data on inpatient TASP models as a suitable solution, describe the essential role of both ID- and non-ID-trained pharmacists in TASPs, and discuss future directions. | Email, web-based systems/conferencing, telephone, shared electronic databases, electronic communication | - Teleconsultation and tele-antimicrobial stewardship (TASP) provide timely access to clinical experts and infrastructure support - Only some demonstrated measurable decreases in antimicrobial consumption, prevalence of multidrug-resistant organisms, or declining C. difficile infection rates. - TASP decreased antimicrobial consumption in some studies, decreased the mean antimicrobial cost and reduced rates of bacterial resistance in some studies.   **-ve:**   - Challenges include asynchronous workflow and indirect communication between the remote and on-site pharmacists. | Technology can increase the number of antimicrobial interventions, improve access to information remotely, and enhance communication amongst care teams. |
| D. Baines, I. K. Gahir, A. Hussain, A. J. Khan, P. Schneider, S. S. Hasan and Z. U. D. Babar (2018) | This paper is a scoping review of the quality and the design of evaluations of mobile health, telehealth, smart pump and monitoring technologies performed in a pharmacy-related setting. | “telehealth” with twenty studies (50.00%): telephone, videoconferencing, electronic monitoring, web-based, shared electronic database  “mobile health” with nine (22.50%): mobile/computer apps, text messaging, electronic monitoring  “monitoring technologies” produced seven (17.50%) studies: electronic monitoring  four papers (10.00%) evaluated “smart pumps.” | - Telehealth technologies focus on communication between patients and pharmacists and extend the knowledge and capabilities of pharmacists, resulting in better patient outcomes. - Failure to adopt new mobile technologies may limit continued professional growth. - Monitoring technologies enable pharmacists to achieve better outcomes for patients, particularly for medication adherence.   **-ve:**   - Most of the evaluations reviewed lacked quality, hence adoption of these new technologies may be a challenge. | Despite the improvements in technology, there is limited evidence on how this translates to real settings and to consumer satisfaction. Most technology driven systems required significant funding and support, particularly those involving latest technology. Rigorous comparative studies are needed to evaluate the effectiveness of different technologies. |
| T. V. Dat, V. L. Tu, N. K. Quan, N. H. Minh, T. D. Trung, T. N. Le, D. Phuc-Vinh, D. T. T. Trinh, L. P. Dinh, H. Y. Nguyen-Thi and N. T. Huy (2022) | To conduct a systematic review of the reported usages, benefits, and limitations of telepharmacy models to further clarify the pros and cons of a telepharmacy model. | Telephone, videoconferencing, text, web-based, remote dispensing, electronic monitoring | - Telepharmacy reduces pharmacist shortage, especially in rural or hard-to-access areas. - It improves the quality of drug use, has no adverse effects on public health, patient safety, and quality of health care and it is not inferior to pharmacies. - Facilitates pharmacists to remotely intervene on drug-related issues, expand the scope of 24-h hospital pharmacies, speed up prescription processing, and reduce order processing time, freeing pharmacists for money-saving or quality-improvement initiatives. - It improves medicine availability during hospital night shifts, to patients in remote areas, and supports remote drug delivery. It saves travel costs, receives higher patient satisfaction, enhances drug safety and effectiveness, decreases preventable adverse reactions, manages chronic diseases, monitor and improves personal clinical indicators, hence improving quality of life. It allows effective collection of medication history for adequate medication reconciliation and improves the speed of doctors' prescribing while optimizing cost-effectiveness - During COVID-19, telepharmacy improves drug-dispensing practices, helping to increase patient access.   **-ve:**   - Challenges include an expected lower-than-average inventory turnover ratio, concern of quality of drug dispensing and medication errors. | Telepharmacy is effective in providing clinical pharmacovigilance related to drugs and managing patients in treatment, thereby increasing the ability to use resources and to save costs. There is potential for telepharmacy to replace or complement pharmaceutical related activities and facilitate future innovation in the health care industry. |
| N. A. Lopez, T. Kerelos and G. Hale (2022) | This project aims to provide a review of pharmacists’ impact during transition of care (TOC) visits utilizing telehealth. | Phone, video conferencing,both teleconferencing software and phone, e-charts, and tablet | - Telepharmacy intervention generally showed improved patient outcomes, lower hospital readmission rates, increased identification of medical discrepancies in patient charts, reduced days from discharge to first outpatient appointment. - Telehealth has a positive impact on TOC and incorporation of pharmacists enhances interventions. - It causes reduction in hospital readmission rates, improvement in patient adherence, and clarification in communication between patients and health care providers.   **-ve:**   - Small sample size, confinement to 1 site or 1 geographic area, or focus on 1 disease state (limits generalizability), lack of randomization, lack of blinding, or lack of a comparator group in some studies, biases | Pharmacist involvement in telehealth monitoring had a positive impact on TOC interventions and overall patient outcomes including decreased hospital readmissions and increased patient medication adherence. |
| T. Melton, H. Jasmin, H. F. Johnson, A. Coley, S. Duffey and C. P. Renfro (2021) | To identify delivery strategies for clinical pharmacy services that use telehealth to serve patients.  A secondary objective was to identify core components of comprehensive medication management (CMM) implemented successfully in telehealth clinical pharmacy services. | Majority of studies (n = 45; 76.3%) utilized a telephone-based intervention, alone or in combination with another mode.  Thirteen studies (22%) used videoconferencing interventions and four studies (6.8%) used web-based interventions  Used alone: Web-based 3 (5.1), Video conferencing 9 (15.3), Telephone 41 (69.5)  Used in combination with telephone: Videoconferencing 2 (3.4), Web-based 1 (1.7), Smart phone application (App) 1 (1.7), Secure email 1 (1.7)  Used in combination with videoconferencing: Smart phone application (App) 1 (1.7) | - Most clinical pharmacy telehealth services were delivered by telephone which could be associated with the lower equipment cost, greater availability amongst patient populations, and ease of use. - Little difference in patients' health outcomes between videoconferencing and telephone.. - Pharmacist-led disease state management programs reported positive outcomes, but lacked heterogeneity, limiting generalisability. Promotes patient access to health care. | Telephone was the most frequently utilized mode of telehealth delivery (unclear reason). Limited use of CMM interventions via telehealth. However, the potential exists to increase CMM uptake and incorporate into intervention designs as health care systems transition to greater telehealth use, particularly after COVID-19. |
