# Supplementary figures and images for: Telehealth Interventions in Pharmacy Practice: Systematic Review of Reviews and Recommendations
Source: J Med Internet Res. 2025 May 7;27:e57129. doi: 10.2196/57129 (PMC12096025; doi:10.2196/57129)

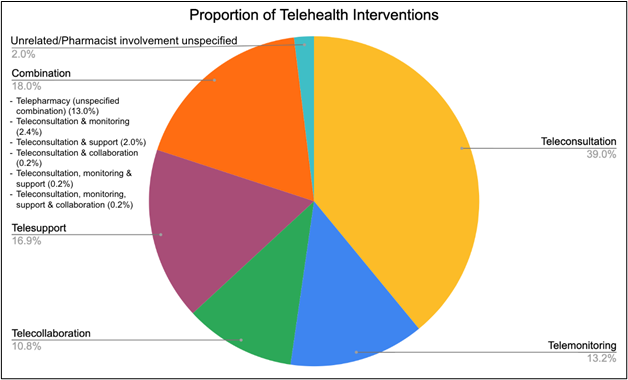

Supplement: Multimedia Appendix 4 [file jmir_v27i1e57129_app4.png]
