# Supplementary material for: Telehealth Interventions in Pharmacy Practice: Systematic Review of Reviews and Recommendations
Source: J Med Internet Res. 2025 May 7;27:e57129. doi: 10.2196/57129 (PMC12096025; doi:10.2196/57129)
Supplement: Multimedia Appendix 5 [file jmir_v27i1e57129_app5.docx]

Multimedia Appendix 4. GRADE-CERQual Table

| **Review findings** | **Studies contributing  (refer to Table 7 for corresponding studies)** | **Methodological limitations** | **Coherence** | **Adequacy** | **Relevance** |
| --- | --- | --- | --- | --- | --- |
| Effect on health outcomes | 1, 5, 6, 8, 10, 11, 12, 14, 16, 17, 18 (11 studies) | 8 no concerns. 2 minor concerns (6, 17). 1 moderate concerns (14). | Minor concerns  Most studies suggested improved health outcomes,  however two reviews indicated no significant differences  in health outcome in some studies between traditional  and telepharmacy groups. | Minor concerns  Two of the reviews included a limited  number of studies (3 and 4 respectively). | No concerns |
| Reduction in hospital readmission rates | 10, 11, 17 (3 studies) | 2 no concerns. 1 minor concerns (17). | No concerns | No concerns | No concerns |
| Improvement in access to healthcare | 3, 4, 5, 6, 8, 9, 13, 18 (8 studies) | 7 no concerns. 1 minor concerns (6). | No concerns | Minor concerns  Two of the reviews included a limited  number of studies (3 and 4 respectively). | No concerns |
| Relieve pharmacist shortage | 3, 4 (2 studies) | 2 no concerns. | No concerns | No concerns | No concerns |
| Impact on cost of healthcare: mixed results | 4, 8, 12, 13, 14, 16, 18 (7 studies) | 6 no concerns. 1 moderate concerns (14). | Moderate concerns  One study showed increased costs with telepharmacy due  to lack of proper reimbursement policies, while three studies  had mixed outcomes with cost savings from eliminating  travel or loss of productivity but greater upfront costs due to  equipment and training needed for telepharmacy. | Minor concerns  One of the reviews included a limited  number of studies (specifically 3 studies). | No concerns |
| Impact on quality: mixed results | 3, 8, 13 (3 studies) | 3 no concerns. | Minor concerns  One review indicated that decreased quality of care was caused  by greater errors and inability to adapt certain assessment tools  for use in telepharmacy. | Minor concerns One of the reviews included a limited  number of studies (specifically 3 studies). | Minor concerns One the definition of quality in one of the studies included  dispensing and filling errors, which overlaps with another  category of the review findings on medication errors. |
| Impact on time: mixed results | 12, 14 (2 studies) | 1 no concerns. 1 moderate concerns (14). | No concerns | No concerns | Minor concerns  Reviews were specific to some types of pharmacists  (eg ICU, tele-antimicrobial stewardship settings), hence  data is not representative of pharmacists in general. |
| Satisfaction with telehealth | 2, 3, 4, 5, 12, 16 (6 studies) | 6 no concerns. | No concerns | Minor concerns  One review did not indicate the number  of articles referenced. | No concerns |
| Medication adherence | 2, 6, 7, 10, 11, 15 (6 studies) | 5 no concerns. 1 minor concerns (6). | No concerns | No concerns | Some reviews were specific to certain patient populations  (eg older adults) or settings (eg outpatient/ambulatory care),  hence data is not representative. |
| Improvement in drug safety | 16 (1 study) | 1 no concerns. | No concerns | Minor concerns  The benefits of telepharmacy to drug safety  was not corroborated by multiple studies. | No concerns |
| Improvement in adverse drug reactions (ADR) | 11, 16 (2 studies) | 2 no concerns. | No concerns | Minor concerns  Reductions of adverse drugs reactions via  telehealth not corroborated by majority of  studies included in both reviews. | No concerns |
| Impact on medication errors: mixed results | 2, 3, 11, 12, 16, 17 (6 studies) | 5 no concerns. 1 minor concerns (17). | No concerns | No concerns | No concerns |
