# Supplementary material for: Telehealth Interventions in Pharmacy Practice: Systematic Review of Reviews and Recommendations
Source: J Med Internet Res. 2025 May 7;27:e57129. doi: 10.2196/57129 (PMC12096025; doi:10.2196/57129)
Supplement: Multimedia Appendix 6 [file jmir_v27i1e57129_app6.docx]

Multimedia Appendix 3. Reasons for Deduction of Scores (Risk Assessment)

|  | **Reasons** | **Effect on Study Quality** |
| --- | --- | --- |
| **Search Strategy** | - Disregard of studies with interventions without express pharmacist involvement (eg. multidisciplinary team) - Not all search terms listed in report - No MesH term/variations, very restricted search term (risk of excluding relevant articles) - Search strategy not stated | -1 (considerable effect)  -1 (minimal effect)  -2 (drastic effect)  -4 (very unsatisfactory) |
| **Screening and Article Selection** | - Potential to exclude relevant articles - Included articles not expressly mentioned - Only RCTs included, potentially excluding relevant articles - Inclusion and exclusion criteria were used but no screening steps (possibility of excluding relevant articles) - No screening stated | -1 (considerable effect)  -1 (minimal effect)  -1 (considerable effect)  -1 (minimal effect)  -4 (very unsatisfactory) |
| **Data Extraction** | - Type of data extracted not mentioned for most articles | -1 (minimal effect) |
| **Data Interpretation** | - Under-reporting of usual care (bias) - Appropriateness of intervention not considered - Applied US telepharmacy definition to other jurisdictions/ no standardization of telepharmacy meaning - Endpoints assessed by patient (not objective, systematic bias) - Conclusions drawn only from patient outcomes, outcomes related to healthcare professionals not mentioned - Low number of studies reviewed, results inconclusive - Studies lacked homogeneity in primary outcomes (unable to draw conclusions) | -1 (considerable effect)  -1 (considerable effect)  -1 (considerable effect)  -2 (drastic effect)  -1 (considerable effect)  -2 (drastic effect)  -1 (considerable effect) |
