# Supplementary material for: Telehealth Interventions in Pharmacy Practice: Systematic Review of Reviews and Recommendations
Source: J Med Internet Res. 2025 May 7;27:e57129. doi: 10.2196/57129 (PMC12096025; doi:10.2196/57129)
Supplement: Multimedia Appendix 7 [file jmir_v27i1e57129_app7.docx]

Multimedia Appendix 5. Corresponding Study Numbers for GRADE-CERQual Assessment

| **Study** | **Number** |
| --- | --- |
| Lowry et al. (2020) | 1 |
| Pathak et al. (2021) | 2 |
| Sarkar et al. (2018) | 3 |
| Baldoni et al. (2019) | 4 |
| Crilly et al. (2020) | 5 |
| Diedrich et al. (2021) | 6 |
| Emadi et al. (2022) | 7 |
| Kane-Gill et al. (2017) | 8 |
| Lobo Borba et al. (2022) | 9 |
| Niznik et al. (2018) | 10 |
| Park et al. (2022) | 11 |
| Strnad et al. (2018) | 12 |
| Unni et al. (2021) | 13 |
| Andrzejewski et al. (2021) | 14 |
| Baines et al. (2018) | 15 |
| Dat et al. (2022) | 16 |
| Lopez et al. (2022) | 17 |
| Melton et al. (2021) | 18 |
